# The Impact of Genetics and Hormonal Contraceptives on the Steroid Profile in Female Athletes

**DOI:** 10.3389/fendo.2014.00050

**Published:** 2014-04-10

**Authors:** Jenny J. Schulze, Jenny E. Mullen, Emma Berglund Lindgren, Magnus Ericsson, Lena Ekström, Angelica Lindén Hirschberg

**Affiliations:** ^1^Division of Clinical Pharmacology, Department of Laboratory Medicine, Karolinska Institutet, Karolinska University Hospital, Stockholm, Sweden; ^2^Department of Women’s and Children’s Health, Karolinska Institutet, Karolinska University Hospital, Stockholm, Sweden

**Keywords:** doping in sports, hormonal contraceptives, genetic polymorphism, UGT2B17, CYP17, testosterone doping, epitestosterone, T/E ratio

## Abstract

The steroid module of the Athlete Biological Passport, the newest innovation in doping testing, is currently being finalized for implementation. Several factors, other than doping, can affect the longitudinal steroid profile. In this study, we investigated the effect of hormonal contraceptives (HC) as well as the effect of three polymorphisms on female steroid profiles in relation to doping controls. The study population consisted of 79 female elite athletes between the ages of 18 and 45. HC were used by 32% of the subjects. A full urinary steroid profile was obtained using World Anti-Doping Agency accredited methods. In addition all subjects were genotyped for copy number variation of *UGT2B17* and SNPs in *UGT2B7* and *CYP17*. Subjects using HC excreted 40% less epitestosterone as compared to non-users (*p* = 0.005) but showed no difference in testosterone excretion. When removing individuals homozygous for the deletion in *UGT2B17*, the testosterone to epitestosterone (T/E) ratio was 29% higher in the HC group (*p* = 0.016). In agreement with previous findings in men, copy number variation of *UGT2B17* had significant effect on female urinary testosterone excretion and therefore also the T/E ratio. Subjects homozygous for the T allele of *CYP17* showed a lower urinary epitestosterone concentration than the other *CYP17* genotypes. It is of great importance that the athlete’s steroidal passport can compensate for all possible normal variability in steroid profiles from women. Therefore, considering the large impact of HC on female steroid profiles, we suggest that the use of HC should be a mandatory question on the doping control form.

## Introduction

Detection of doping with endogenous steroids, such as testosterone, has been and continues to be a challenge. To overcome the problem of separating testosterone doping from endogenous testosterone the ratio between the glucuronides of testosterone and epitestosterone (T/E) is used. This T/E ratio was introduced in doping tests with an authorized upper limit of 4. Interestingly, the mean T/E ratio in Caucasian men is approximately 1 (1), whereas in Asians, the mean ratio is considerably lower (2).

Glucuronidation of androgens by UDP-glucuronosyltransferases (UGTs), i.e., phase II metabolism, is the major route for androgen inactivation and excretion (3–6). UGT2B7 has been identified as the enzyme responsible for epitestosterone conjugation (7, 8) whereas testosterone is a poor substrate for this enzyme. Testosterone is mainly conjugated by UGT2B17 and to a minor extent by UGT2B15. We have recently shown that testosterone glucuronidation activity in the liver is significantly higher in men homozygous for the insertion (*ins*/*ins*) of *UGT2B17* than in women with the same genotype (9).

We have also shown that the ethnic disparity in the T/E ratio is strongly associated with a deletion polymorphism in the *UGT2B17* gene (10). Individuals homozygous for this deletion (*del*/*del*) may not reach a T/E ratio of 4 when doped with testosterone (11). The deletion polymorphism is much more common in East Asian populations as compared to Caucasians and Africans (12). There are also individuals that have naturally high T/E ratios due to decreased excretion of epitestosterone. In males, part of this low epitestosterone excretion can be explained by a promoter polymorphism in the *CYP17* gene (13), resulting in 64% higher T/E ratios in men homozygous for the T allele (14). However, this polymorphism in relation to epitestosterone excretion has not been studied in women.

Women show a greater individual variation in T/E ratio than men due to concentrations near the detection limit for the method of analysis as well as variations during the menstrual cycle (15). Furthermore, the use of hormonal contraceptives (HC) has been suggested to suppress the production of epitestosterone and thus lead to an increase in the T/E ratio (16). In Sweden, a study has shown that almost half of female elite athletes are using HC, a frequency comparable to the general population of the same age group (17). HC consist of a progesterone derivative (progestogens) or a combination of a progestogen and synthetic or natural estrogen. Their main mechanism of action is inhibition of ovulation. Both progesterone and estrogen are negative regulators of the hypothalamic–pituitary–gonadal (HPG) axis, meaning that an increase in these sex hormones results in a decrease in gonadotropin releasing hormone (GnRH), luteinizing hormone (LH), and follicle stimulating hormone (FSH).

In 1994, it was shown that between-subject variation can be removed in doping tests by using a series of measurements obtained from the same individual (18). Six years later the concept of the Athlete Biological Passport (ABP) was proposed. The ABP uses a longitudinal approach where an individual’s previous results are logged and compared to the new results (19). The hematological module of the ABP, used to detect blood doping, has successfully been in use since 2009, while the module for steroid doping is currently being finalized for implementation.

In this study, we investigated how three polymorphisms and HC affect the steroid module of the ABP. Previously, there has only been a few studies investigating the effect of genetic variation and HC on female steroid profiles in relation to doping controls. With the implementation of the steroid module of the ABP, the sensitivity of doping tests has improved. The improved sensitivity requires increased knowledge of how external factors influence the steroid profile. More knowledge in this area is of great importance for correct interpretation of female steroid profiles using the ABP program.

## Materials and Methods

### Study population

The study population included 57 female Olympic level athletes who planned to participate in the up-coming summer or winter Olympic Games (either Olympic team members or members within the programs of the Swedish Olympic Committee). The mean age of these subjects was 26 years with a range from 18 to 45. Information about HC use was collected and a written consent was obtained from all subjects.

In addition, 22 elite female athletes, whom have given consent for research on their blood and urine samples in their anonymous doping control forms, were included in the study. The medical information on the doping control form was used to gather information on hormonal contraceptive use.

Out of the 79 female athletes, 25 (32%) were using HC. Two women were using progestogen only contraceptives (birth control implant or desogestrel) while the remaining 23 were using combined oral contraceptives. Sixteen subjects (68% of those on HC) used progestogen dominated HC of the second generation (levonorgestrel/ethinylestradiol or norethisterone/ethinylestradiol), while 8 subjects (32% of those on HC) used estrogen dominated HC of the third and fourth generation [norgestimate/ethinylestradiol, drospirenone/ethinylestradiol or etonogestrel/ethinylestradiol (vaginal ring)].

### Measurement of urinary steroids

Urinary levels of testosterone, epitestosterone, androsterone (A), etiocholanolone (Etio), 5α-androstan-3α,17β-diol (5α-diol), and 5β-androstan-α,17βdiol (5β-diol) were determined following the validated screening method of the World Anti-Doping Agency (WADA) accredited doping laboratory. This method uses gas chromatography–mass spectrometry (GC/MS) essentially as described by Chung et al. (20), with minor modifications as previously described (21). All urinary values are expressed as the unconjugated [typically <8% of glucuronide fraction (22)] plus the glucuronide conjugated fraction. The effect of urine dilution was adjusted for by normalizing the samples to a specific gravity of 1.020.

### Copy number analysis of UGT2B17

DNA was extracted from the whole blood samples using QIAamp^®^ DNA Blood Mini kit (Qiagen). Triplicates of 10 ng/μL DNA-samples were used to genotype for the *UGT2B17* deletion polymorphism as we have previously described (14). This genotyping was achieved by using 7500 Fast Real-Time PCR systems with albumin as internal control as described by Schaeffeler et al. (23).

### UGT2B7 and CYP17 SNP analyses

Genotyping assays from Applied Biosystems (Foster City, CA, USA) were used to genotype for *UGT2B7 H^268^Y* and the T > C substitution in the promoter region of *CYP17A1*. For *CYP17* a premade 20× genotyping mix was used (c_2852784_30) with 2× TaqMan Universal PCR Master mix and 1 μL DNA sample for a total of 15 μL. For the *UGT2B7* genotyping a mix was prepared with 2× TaqMan Universal PCR Master Mix, 0.6 μM forward and reverse primer (forward: AGC TGA CGT ATG GCT TAT TCG AA, reverse: GGG TTT GGC AGG TTT GCA), 0.1 μM of both probes each containing a different allele (FAM-TTC AGT TTC C*AT* ATC CAC TCT-MGB, VIC-TTC AGT TTC C*TC* ATC CAC TCT-MGB) and 3 μL DNA sample per 15 μL reaction. Thermal cycling followed a protocol for activation at 95°C for 10 min; 40 cycles of denaturation at 96°C for 15 s, annealing/elongation at 60°C for 1 min, and lastly a hold at 4°C.

### Data evaluation

As most of the data were not normally distributed, the median with range is given for all data, even in the few cases where the data were normally distributed. Statistical analyses were performed according to distribution with Student’s two-tailed *t*-test or the Mann–Whitney *U*-test using GraphPad PRISM 6.0^®^ (GraphPad, San Diego, CA, USA).

## Results

### Influence of hormonal contraceptives

**Figure 1:**
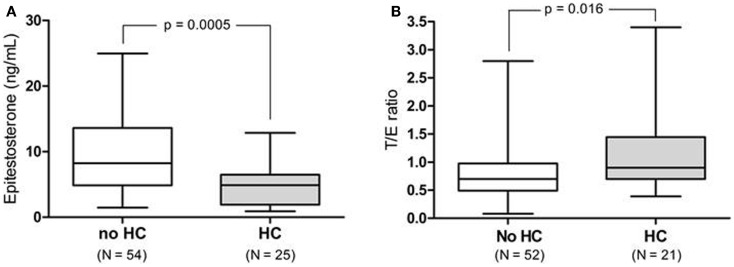
**(A)** Urinary epitestosterone concentration in elite female athletes not taking hormonal contraceptives (non-HC) as compared to those on HC. **(B)** T/E ratio for the same athletes after removing the *UGT2B17 del*/*del* individuals (*N* = 6).

Female athletes using HC excreted epitestosterone at a significantly lower rate [4.9 (range: 0.90–12.9) ng/mL] than females who did not use HC [8.2 (range: 1.5–25.0) ng/mL, *p* = 0.0005, Figure 1A]. When removing the *UGT2B17 del*/*del* individuals (*N* = 6) the T/E ratio was significantly higher in HC-users as compared to non-users [0.9 (range: 0.39–3.4) and 0.7 (range: 0.08–2.8) ng/mL, respectively, *p* = 0.016, Figure 1B]. There was no difference in urinary testosterone levels in the HC group compared to the non-HC group. There was also no difference in the A/Etio ratio nor the 5α-diol/5β-diol ratio between the HC-users (0.80 and 0.23) and the non-users (0.76 and 0.32). The A/E ratio was significantly higher in the HC group (491, range: 59.4–994) compared to the non-HC group (247, range: 63.1–589) as a consequence of the low epitestosterone levels in hormonal contraceptive users (*p* < 0.0001). The data are presented in Table 1.

**Table 1:** **Comparison between urinary steroid profiles for the non-HC and HC group as well as urinary steroid profiles for different genotypes**.

All subjects	Non-HC	HC	*p*-Value
*N* (%)	54 (68.4%)	25 (31.6%)	
Testosterone (ng/mL)	4.1 (1.2–19.4)	4.5 (0.74–14.4)	NS
Epitestosterone (ng/mL)	8.2 (1.5–25.0)	4.9 (0.9–12.9)	0.0005
T/E ratio	0.7 (0.08–2.8)	0.9 (0.39–3.4)	0.016
A/Etio ratio	0.76 (0.22–1.9)	0.80 (0.41–2.0)	NS
5α-diol/5β-diol ratio	0.32 (0.06–1.0)	0.23 (0.08–0.68)	NS
A/E ratio	247 (63.1–589)	491 (59.4–994)	<0.0001

**UGT2B17**	**Del/Del**	**Ins/Del + Ins/Ins**	***p*-Value**

*N* (%)	6 (7.6%)	73 (92.4.0%)	
Testosterone (ng/mL)	0.66 (0.46–1.5)	5.4 (0.74–19.4)	<0.0001
Epitestosterone (ng/mL)	4.9 (1.8–21.5)	6.6 (1.2–25.0)	NS
T/E ratio	0.15 (0.09–0.3)	0.8 (0.08–3.4)	0.0002
A/Etio ratio	1.1 (0.42–2.0)	0.77 (0.21–1.9)	NS
5α-diol/5β-diol ratio	0.99 (0.45–1.8)	0.32 (0.06–1.0)	0.0018
A/E ratio	512 (179–994)	278 (59.4–849)	NS

**UGT2B7 no HC**	**HH + H/Y**	**YY**	***p*-Value**

*N* (%)	38 (71.7%)	15 (28.3%)	
Testosterone (ng/mL)	4.7 (1.2–13.7)	6.7 (2.1–19.4)	NS
Epitestosterone (ng/mL)	6.9 (1.5–25.0)	11.2 (4.5–10.9)	NS
T/E ratio	0.70 (0.08–2.8)	0.59 (0.11–1.6)	NS
A/Etio ratio	0.78 (0.22–1.8)	0.77 (0.22–1.9)	NS
5α-diol/5β-diol ratio	0.32 (0.09–1.0)	0.32 (0.06–0.64)	NS

**UGT2B7 HC**	**HH + H/Y**	**YY**	***p*-Value**

*N* (%)	22 (88.0%)	3 (12.0%)	
Testosterone (ng/mL)	4.3 (0.74–14.4)	11.1 (8.9–13.3)	Too few
Epitestosterone (ng/mL)	3.9 (0.9–11.3)	6.2 (5.1–12.9)	0.059
T/E ratio	0.9 (0.39–3.4)	1.2 (0.7–1.6)	Too few
A/Etio ratio	0.78 (0.41–1.5)	0.86 (0.78–2.0)	NS
5α-diol/5β-diol ratio	0.26 (0.08–0.68)	0.23 (0.21–0.25)	Too few

**CYP17A1 non-HC**	**TT**	**CT + CC**	***p*-Value**

*N* (%)	25 (47.2%)	28 (52.8%)	
Testosterone (ng/mL)	6.6 (2.1–13.7)	4.7 (1.2–19.4)	NS
Epitestosterone (ng/mL)	8.6 (2.6–22.8)	7.9 (1.5–25.0)	NS
T/E ratio	0.70 (0.30–2.6)	0.6 (0.08–2.8)	NS
A/Etio ratio	0.80 (0.22–1.4)	0.76 (0.32–1.9)	NS
5α-diol/5β-diol ratio	0.32 (0.09–1.0)	0.34 (0.06–0.92)	NS

**CYP17A1 HC**	**TT**	**CT + CC**	***p*-Value**

*N* (%)	10 (40.0%)	15 (60.0%)	
Testosterone (ng/mL)	2.4 (0.74–9.4)	6.2 (2.3–14.4)	0.017
Epitestosterone (ng/mL)	2.2 (0.91–5.9)	5.6 (1.6–12.9)	0.0036
T/E ratio	0.99 (0.50–3.4)	0.80 (0.39–1.8)	NS
A/Etio ratio	0.76 (0.44–1.2)	0.80 (0.41–2.0)	NS
5α-diol/5β-diol ratio	0.38 (0.16–0.49)	0.21 (0.08–0.68)	NS

*The results are shown as median values with the range in parenthesis (all values are corrected for by specific gravity). NS, non-significant; A, androsterone; Etio, etiocholanolone; 5α-diol, 5α-androstan-3α,17β-diol, 5β-diol, 5β-androstan-α,17βdiol; E, epitestosterone*.

### Polymorphisms in UGT2B17, UGT2B7, and CYP17 and impact on urinary androgens

The allele frequencies of the genotyping analysis are shown in Table 2. It should be noted that all of the subjects are Swedish; however, some of them may have other ethnic descent than Caucasian. The genotyping results are in line with previous published allele frequencies in Caucasians (14, 24).

**Table 2:** **Allele frequencies of the three investigated polymorphisms shown as total number of athletes with the specific genotype and percentage of the whole study group**.

UGT2B17	Del/Del	Ins/Del	Ins/Ins
	6 (8.3%)	40 (58.3%)	22 (33.3%)

**UGT2B7**	**HH**	**HY**	**YY**

	16 (20.5%)	44 (56.4%)	18 (23.1%)

**CYP17A1**	**TT**	**CT**	**CC**

	35 (44.9%)	31 (39.7.6%)	12 (15.4%)

#### Influence of the UGT2B17 deletion polymorphism

The *UGT2B17* genotype had a large impact on the urinary testosterone levels and the T/E ratio. As there were only six *del*/*del* individuals, two in the non-HC group and four in the HC group the urinary steroid levels and ratios were only calculated for the whole group (Table 1). There were no significant differences between the *ins*/*del* and the *ins*/*ins* group in testosterone levels or T/E ratio in line with previous published results for female athletes (9). The 5α-diol/5β-diol ratio was significantly higher in the *del*/*del* group as compared to the combined *ins*/*ins* and *ins*/*del* group [0.99 (range: 0.45–1.8) and 0.32 (range: 0.06–0.64), respectively, *p* = 0.0018].

#### Influence of the UGT2B7 H268Y polymorphism

As a previous study showed that the YY genotype had higher glucuronidation activity than the HH and HY genotype, the study subjects were divided into HH + HY and YY groups for increased statistical power (25). There was however only three YY individuals in the HC group, which made it difficult to interpret the data in this group and no *p*-values could be calculated. One of the YY individuals also had a double deletion of the *UGT2B17* gene, and had to be excluded from the testosterone, T/E, and 5α-diol/5β-diol ratio calculations. There was a tendency toward higher epitestosterone levels in the YY group both for users and non-users of HC (Table 1).

#### Influence of the CYP17 promoter polymorphism

**Figure 2:**
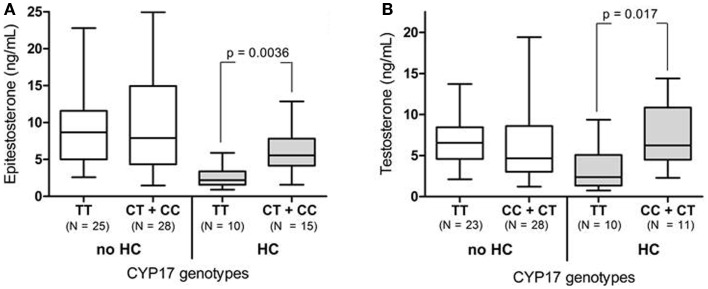
**(A)** The urinary epitestosterone concentration for elite female athletes divided in *CYP17* genotypes and HC use. The CC and CT genotypes were combined to increase statistical power. **(B)** The urinary testosterone concentrations after removing the *UGT2B17 del*/*del* individuals.

For increased statistical power the CC and CT group was combined for the *CYP17* genotyping analysis (Table 1). Among the HC-users, the TT genotype was significantly associated with lower epitestosterone levels [2.2 (range: 0.91–5.9)] compared to the CT + CC genotype [5.6 (range: 1.6–12.9)] (*p* = 0.0036, Figure 2A; Table 1). After removing the *del*/*del* individuals the HC-users homozygous for the TT genotype also had significantly lower testosterone levels as compared to the CT + CC genotypes [2.4 (range: 0.74–9.4) and 6.2 (range: 2.3–14.4), respectively, *p* = 0.017, Figure 2B].

## Discussion

In this study, we show that use of HC has an impact on epitestosterone excretion, no effect on testosterone excretion and thus significant effects on the T/E ratio. Urinary epitestosterone levels were 40% lower in the HC-users when compared to non-users. A 29% increase in T/E ratio could be discerned first after the individuals homozygous for the deletion polymorphism in *UGT2B17* were excluded. Furthermore, our results confirm a strong link between *UGT2B17* deletion polymorphism and urinary testosterone levels in our female study group (9, 26). Here we show for the first time a link between a SNP in the promoter region of *CYP17* and epitestosterone excretion in females. The subjects homozygous for the T allele showed lower epitestosterone levels than subjects with the CT and CC genotype.

Studies have shown that females display a larger intra-individual variation in T/E as compared to men and that the fluctuation of T/E in women varies throughout the menstrual cycle (16, 27, 28). Mareck-Engelke et al. measured urinary steroid profiles in four women during two menstrual cycles with and without oral contraceptives. They showed that the epitestosterone levels were lower and varied less over the menstrual cycle in women using HC. Without HC, the epitestosterone excretion seems to increase during the last 2 weeks of the menstrual cycle, with a large inter-individual variation (15). We were able to confirm the findings of Mareck-Engelke’s that epitestosterone excretion decreases with the application of HC. Our study population was too small to further investigate the specific impact of estrogenic and progestogen dominated contraceptives, but one might speculate that progestogen dominated HC have a more profound effect on epitestosterone excretion since progestogens are known to repress LH to a larger extent than estrogens (29).

It has been shown that oral contraceptives reduce both ovarian and adrenal androgen production (30–33). The studies showed that serum testosterone, free testosterone, and serum dehydroepiandrosterone (DHEA) were significantly lower in HC-users. None of these studies have, however, analyzed serum epitestosterone concentrations. Unfortunately, we do not have serum data from our study subjects to investigate changes in serum concentrations. A possible explanation for the reduction of urinary epitestosterone while testosterone excretion stays unchanged is that 30% of epitestosterone is excreted unchanged (apart from being conjugated with glucuronic acid) as compared to only ~1% of testosterone (34). Thus, an equal change in (T/E) production will result in a 30 times larger change in epitestosterone than testosterone excretion.

The CYP17 enzyme mediates central activities and functions in human steroidogenesis in the adrenal gland, ovaries, and testes (35, 36). The *CYP17* promoter polymorphism has been extensively studied. The CC (A2) variant was hypothesized to create an additional Sp1 site in the promoter and thereby increase the transcription of this gene (13), however activity studies have not been able to confirm this finding (37). The theory is, however, consistent with our findings in this and previous work (14) where the C-allele is associated with increased levels of epitestosterone. We were able to confirm our previous finding in men (14) that the C-allele is associated with significantly higher urinary epitestosterone concentrations. This was only significant among the HC-users. This may be due to the large variation in epitestosterone levels during the menstrual cycle in non-HC-users. If urine had been collected on the same day of the menstrual cycle (preferably in the beginning) the difference may also have been shown in the non-HC group. However, during urine collection for doping tests, as in this study, the status of the menstrual cycle will be an unknown factor. The C-allele was also associated with higher testosterone levels in HC-users. A better controlled study in a larger group of females is needed to confirm these findings. Previous findings are inconsistent with some studies supporting higher levels of androgens, estrogens, and DHEA while others showed no difference (38). This, and our previous work (14), is to our knowledge the first to associate this polymorphism with steroid levels in urine.

The UGT2B7 enzyme is known to be the main catalyst of epitestosterone conjugation (8). Androsterone and androstanediol are also known substrates for this enzyme. A polymorphism H268Y has been described in this gene, resulting in an amino acid change within the substrate binding site (39). The YY variant has been shown to be associated with significantly higher levels of androstanediol glucuronides in serum (25). We were unable to find any associations with this polymorphism and urinary steroid levels (Table 1). The impact of genetic polymorphisms on the steroid profile must be carefully interpreted since the study group is small and the variation caused by the menstrual cycle in the non-HC-users is large.

In addition to T/E ratio, other steroid metabolite ratios (e.g., A/Etio, 5α-diol/5β-diol, and A/E) are often used as biomarkers in the evaluation of a steroid profile. The ratio of A/Etio and 5α-diol/5β-diol does not seem to be affected during HC application, which is important for doping control purposes. However, the 5α-diol/5β-diol ratio, an important marker for dihydrotestosterone doping (40), is affected by the *UGT2B17* deletion polymorphism (12, 26, 41) as 5β-diol is mainly glucuronidated by UGT2B17 (8). We and others have reported that A/E ratio is a sensitive marker for testosterone doping. The A/E ratio is, unlike the T/E and 5α-diol/5β-diol ratio, unaffected by the *UGT2B17* deletion polymorphism (42, 43). This ratio is however markedly affected by the use of HC. As this ratio as well as the T/E ratio is affected by HC, we share Mareck-Engelke’s concern that there will be major changes when starting or stopping the use of HCs.

The up-coming implementation of the steroidal passport will improve sensitivity in doping tests for steroids. This sensitivity is however, not limited to doping. Factors such as genetics or drugs use, can also affect the measured biomarkers. Genetic and other influences on the steroid profile have not been extensively studied in women. It is paramount that the athletic steroidal passport program can compensate for all possible standard variability in steroid profiles from women. In this study we show, for the first time, that HC as well as polymorphisms in *UGT2B17* and *CYP17* affect the female steroid profile. We suggest including information on HC use as a standard question on the doping control form for urine tests. This information should include whether an athlete recently started or stopped taking HC as well as the duration for which she has taken the current brand.

## Conflict of Interest Statement

The authors declare that the research was conducted in the absence of any commercial or financial relationships that could be construed as a potential conflict of interest.
